# Considering landscape heterogeneity improves the inference of inter-individual interactions from movement data

**DOI:** 10.1186/s40462-025-00567-0

**Published:** 2025-06-12

**Authors:** Thibault Fronville, Niels Blaum, Florian Jeltsch, Stephanie Kramer-Schadt, Viktoriia Radchuk

**Affiliations:** 1 Department of Ecological Dynamics, Leibniz Institute for Zoo and Wildlife Research (IZW), Alfred-Kowalke-Straße 17, 10315 Berlin, Germany; 2 Department of Ecology, Technische Universität Berlin, Rothenburgstr. 12, 12165 Berlin, Germany; 3 Department of Plant Ecology and Nature Conservation, University of Potsdam, Am Mühlenberg 3, 14476 Potsdam, Germany

**Keywords:** Statistical methods, Movement data, Inter-individual interactions, Physical environmental, Habitat heterogeneity, Collinearity, Confounding

## Abstract

**Background:**

Animal movement is influenced by both the physical environment and social environment. The effects of both environments are not independent from each other and identifying whether the resulting movement trajectories are shaped by interactions between individuals or whether they are the result of their physical environment, is important for understanding animal movement decisions.

**Methods:**

Here, we assessed whether the commonly used methods for inferring interactions between moving individuals could discern the effects of environment and other moving individuals on the movement of the focal individual. We used three statistical methods: dynamic interaction index, and two methods based on step selection functions. We created five scenarios in which the animals' movements were influenced either by their physical environment alone or by inter-individual interactions. The physical environment is constructed such that it leads to a correlation between the movement trajectories of two individuals.

**Results:**

We found that neglecting the effects of physical environmental features when analysing interactions between moving animals leads to biased inference, i.e. inter-individual interactions spuriously inferred as affecting the movement of the focal individual. We suggest that landscape data should always be included when analysing animal interactions from movement data. In the absence of landscape data, the inference of inter-individual interactions is improved by applying ‘Spatial+’, a recently introduced method that reduces the bias of unmeasured spatial factors.

**Conclusions:**

This study contributes to improved inference of biotic and abiotic effects on individual movement obtained by telemetry data. Step selection functions are flexible tools that offer the possibility to include multiple factors of interest as well as combine it with Spatial+.

**Supplementary Information:**

The online version contains supplementary material available at 10.1186/s40462-025-00567-0.

## Introduction

Animal movement is a fundamental behavioural process that results in a change of spatial locations of an individual and has important implications for its survival and reproduction, with consequences on the population and community level. Different types of movement can be distinguished, such as foraging movement, migratory movement, dispersal, and nomadic movement [6, 35, 44]. The decision of how, where and when to move is influenced by both the physical environment of an animal and its social environment [10, 35, 48]. The physical environment may consist of habitat features that offer resource and shelter, but also structures like barriers that hinder movement. The social environment consists of con- and heterospecifics and exchange of information with them. The social environment may be beneficial but also disadvantageous. Animals might be attracted to other individuals due to the benefits of sociality like social grooming, reduced predation risk, increased foraging efficiency or access to social information [54]. Or they may avoid other individuals to reduce the risk of pathogen transmission [34], competition [29] and predation [17, 28, 31]. Importantly, the physical environment and social environment are not independent of each other as the physical environment might facilitate or constrain animal interactions. Indeed, a correlation in the movement trajectories of several individuals might emerge from gathering at the same resource. For example, mammal individuals in arid ecosystems regularly come to water holes and are primarily interested in water as resource [53]. However, when their movement trajectories would be analysed without explicitly considering water as a resource, it may seem as if individuals interact with each other, while they are foremost interested in the water resource. On the other hand, barriers might prevent them from meeting. Populations might get separated due to constraints on their movement introduced by habitat fragmentation [5, 27, 40]. Thus, the physical environment in which animals move will fundamentally shape the patterns of social interactions. Identifying whether correlated movement paths of two (or more) individuals arise from interactions between them or whether they are the result of their physical environment is important for understanding the cause of animal movement and behaviour.

A rapid development of tracking technologies in recent years allowed the collection of high-resolution data on multiple simultaneously moving individuals. This, in turn, motivated the development of several methods to infer interactions among moving animals [9, 18, 32, 37, 45, 47], which now open the possibility to explore how animals move relative to one another. For example, dynamic interaction indices [33] are commonly used to analyse interactions between two individuals, while step selection functions can be used to identify animals’ preference towards landscape features or even other individuals [45].

Here, we assess the ability of three methods to correctly detect whether animal movement paths emerge from inter-individual interactions or if they are simply the by-product of individuals responding to the same environmental features. We focused on three statistical methods extensively tested in Fronville et al. [18]: one commonly used index of dynamic interaction (DI—Dynamic interaction index) that is implemented within the ‘Wildlife DI’ R package [32] and two methods that are based on step-selection functions (SSF): one uses as a covariate the occurrence distribution of the other moving individual [45], herewith referred to as SSF-OD) and the other one uses the distance to the other moving individual(s) [43], herewith referred to as SSF-DIST). All three methods are used to estimate interactions from movement trajectories, i.e. time series of location estimates collected on at least two simultaneously moving individuals. Both SSF-based approaches also can account for other covariates (e.g. environmental data) when assessing inter-individual interactions, allowing us to investigate how these movement trajectories are shaped by resources and obstacles in the environment [36]. Furthermore, we tested whether the bias of unmeasured spatial factors on the social interactions can be reduced or even fully eliminated by applying a method called ‘Spatial+’ in combination with the SSFs [12]. ‘Spatial+’ removes the effect of space on the considered covariate (e.g. the occurrence distribution of the other moving individual or the distance to it) and thereby reduces the bias in the effect estimates.

**Fig. 1:**
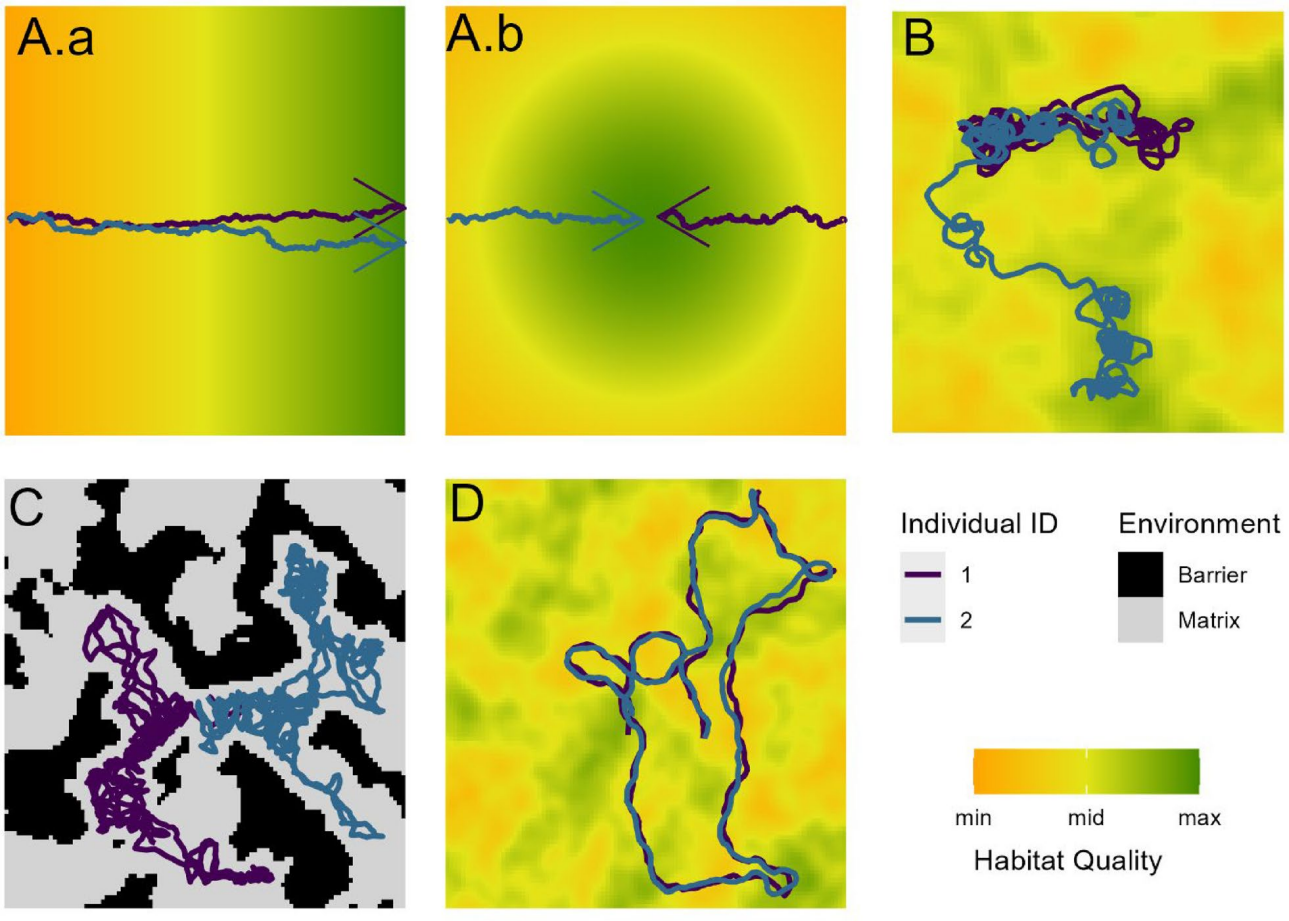
Schematic representation of the four scenarios: Panels **A**–**C** show correlated movements of two individuals caused by habitat quality and barriers (no social interaction). **A.a** moving next to each other along a linear resource gradient, **A.b** moving from opposite initial locations towards the resource in the centre. **B** moving in a realistic landscape where resources are clustered in space. **C** Movement in a homogeneous matrix with barriers randomly scattered in the landscape blocking the movement of individuals. In Panel D the two individuals are attracted to each other and move as a group in the landscape, irrespective of habitat quality or other features. The violet and blue arrows depict the movement path of two individuals, the direction shown by the arrow. In panel A, B and D each grid cell reflects habitat quality that ranges from zero (orange) to one (green). Panel C shows the matrix in grey and the barriers, which are avoided by the individuals, in black

To test the methods and to investigate the possible risks of neglecting the effects of physical environmental features when analysing interactions between moving animals, we simulated movement data with a spatially-explicit agent-based model (ABM, [22, 49]) introduced in Fronville et al. [18]. Using an agent-based and spatially-explicit modelling approach provided full system knowledge and allowed us to generate different landscape scenarios with which the simulated individuals could interact. We simulated four landscape scenarios: in three of them (Fig. 1a–c) two individuals do not interact with each other, but their movement is affected in the same way by their physical environment, leading to resulting correlated movement trajectories; in the fourth scenario (Fig. 1d) two individuals interact exclusively with each other and are not affected by their physical environment. In this study, the physical environment consists of resources (gradient of habitat quality) that the individuals are attracted to, or barriers (e.g. rivers, human-made structures) that hinder their movement. In scenarios A and B, the individuals are attracted to the same resources and they move along a gradient of habitat quality (scenario A) and within a patchy landscape where resources are clustered (scenario B). In scenario C the individuals move in a homogeneous matrix that is intercepted by barriers, which are randomly scattered in the landscape and can hinder or “guide” individual movement. The landscapes are built in such a way that a correlation in movement trajectories of two individuals arises either because both individuals are attracted to the same habitat quality (in scenarios A and B) or because the movement of both individuals is constrained by barriers (in the scenario C) leading to their “enforced” correlation.

Since DI does not allow accounting for the effect of environmental predictors when assessing interactions and in case of spatially correlated trajectories results in values close to “1” [33], we expect that it will falsely detect interactions between both individuals when their movement is, in fact, affected by the resource only. When the physical environment is included as a covariate in SSF, we expect the SSF-based approaches to correctly detect that the correlated movement trajectories of the two individuals emerge from the effect of their physical environment. Yet, in case the relevant physical environmental covariate is not considered in the analyses, either because the researchers do not have access to relevant environmental descriptors or because they do not expect environment to strongly affect individual movement, both these methods will, similarly to DI, falsely detect interactions between the individuals. However, we expect that applying spatial+ in such cases will partial out the spatial dependence and remove the spurious interaction effects.

## Methods

### Simulation of movement trajectories

In the Agent-Based Model (ABM) two individuals move according to a biased correlated random walk in discrete time. At each time step a turning angle and a step length is drawn from the respective distributions, as detailed below. To generate the turning angles, we used the von Mises distribution with a concentration parameter of four (strong directional persistency). For the step lengths we used the gamma distribution with the parameters scale = 0.15 and shape = 6. There are two different mechanisms that generate the biased movement: either through an attraction towards another moving individual via distance or through the selection of the environmental surroundings via resource/ habitat quality. This selection is done by a step selection function [46]. Both biases in movement direction are considered as interactions (with conspecifics and with the environment, respectively) in our simulation. The individuals are moving within an area of a fixed size with reflecting borders, whereas the area modelled is so large that encounters with borders are rare. Four different scenarios are then devised in which the individuals interact with their environment (Fig. 1a-d). The detailed model description follows the Overview, Design, concepts, Details (ODD) protocol by Grimm et al. [22] and updated [23] (Supporting information).

#### Interaction with environmental surroundings

Individuals can interact with their environmental surroundings while moving (Fig. 1a–c), i.e. are attracted (selection towards a resource) or avoid (barriers); or do not interact (i.e. purely perform correlated random walk). In these scenarios both individuals are not directly interacting with each other, yet their resulting movement trajectories are correlated due to the individuals responding to the environmental surroundings in the same way.

We generated three scenarios of interactions of both individuals with environments (Fig. 1a–c). For two scenarios, we used a grid-based environment in which each cell reflects habitat suitability. The values of habitat suitability range from zero to one. In the first scenario A, we model a gradient of habitat suitability with habitat suitability increasing linearly from the west to the east of the simulated area (Fig. 1A.a) or increasing linearly from the borders towards the centre (Fig. 1A.b). For the second scenario B, the grid cells were assigned with a value generated with the Perlin noise function. The Perlin noise is used to generate not completely random values [39] and is helpful to create procedurally generated landscapes. This produces patches of grid cells with values similar to each other, which gives the appearance of more naturally clustered patchy landscapes compared to full randomness (Fig. 1b). For the scenarios A & B, the moving individuals evaluate the cells within their perceptual range and bias their movement towards the cell with the highest value. The Perlin noise function is also used to generate the barriers for the third scenario C, which blocks the path of the individuals (Fig. 1c). In this case, all the cells with values higher than the threshold (ranges between 0.5 and 1 depending on the percentage of barriers) were assigned as barriers (set value to zero) and avoided by the individuals. The individuals do not express any preference towards a particular grid cell other than that they will not enter the barrier cells. This is accomplished by only picking randomly generated steps that land outside the barrier. For the scenario C the proportion of barriers in the landscape was continually varied from 0–50% of the total landscape area with 0.5% steps, resulting in 100 barrier landscapes. Additionally, 20 repetitions of a simulation with 70% barriers in the landscape were run for scenario C to imitate a scenario with extreme fragmentation in the landscape.

#### Interaction with other individuals

In the scenario D both individuals are attracted towards each other but do not interact with their environmental surroundings (Fig. 1d). They both move according to a correlated random walk while they express a positive bias towards each other (higher likelihood of selecting steps closer to the other individual) resulting in them moving as a pair within the environment. The simulation was run for 20 repetitions, each with a newly generated landscape structure.

### Statistical methods for inferring interactions from movement data

For a comprehensive description and a summary table of the statistical methods employed in this study, please refer to the Supplementary Methods section.

#### Dynamic interaction index (DI)

The DI index measures the cohesiveness of simultaneous movement vectors with respect to two independent components of movement: distance (also called displacement) and direction (DI; [32]). Values for DI range from -1 to 1, where negative values correspond to repulsive movement paths (opposite direction) and positive values indicate cohesive movement paths (in same direction). Values around 0 indicate neutral movement. The p-values are computed following Benhamou et al. [8]. The associated p-value is generated from a permutation test (IAB)—the actual movement is compared to randomized independent movement.

#### SSF-based approaches

The SSFs compare observed movement steps of a focal individual to available steps in terms of certain covariates, which allows to quantify a preference for these covariates (usually environmental variables) [16]. The step lengths and turning angles for the available steps were drawn from a gamma and von Mises distribution, respectively, of which the parameter estimates were obtained from the simulated steps of our individuals. We generated 20 available steps per simulated step. The estimation of selection coefficients was done using a conditional logistic regression, allowing to compare each simulated step to a different set of available steps. In other words, the available steps depend on the location and movement characteristics of the individual (temporally varying set of available steps). Positive coefficients indicate attraction and negative coefficients indicate repulsion, while zero indicates no detectable preference for the covariates. The SSF-based approach by Schlaegel et al. [45] uses as a covariate dynamic occurrence distributions (OD) of other individuals [15] and is referred to as SSF-OD approach in this study. The occurrence distribution can be calculated from multiple individuals, but in this study the OD was calculated from only one individual. The second SSF-based approach we use is a modification of SSF-OD that, instead of the occurrence distribution, uses distances between individuals (DIST) as covariates in SSFs [43]. We refer to this approach as SSF-DIST.

#### Spatial+

Spatial confounding (collinearity/non-independence between the covariate of interest and unmeasured spatial effect) is often an issue when working with spatial data and can lead to biases in the estimated covariate effects. Spatial+ [12] is used to reduce this bias, by reducing the spatial dependence of the covariate using penalized thin plate regression splines, which is done in two steps. Firstly, the spatial dependence is regressed by using the covariate of interest as response variable and the thin plate spline of coordinates as independent predictor. In the second step, the residuals obtained in the first step as well as the spline fit are used as covariates (corrected covariate) in the SSF. This approach was only used for the SSFs and not DI, because no covariates can be included as predictors in DI.

### Evaluating method performance

We assessed the performance of the methods by focusing on the power of the methods to detect true interactions and by evaluating type 1 error for wrongly detected interactions (false positives). We used the *p-values* of each method to validate its outcome to be significant (< 0.05) or not. The proportion of correctly estimated interactions was then used as a metric indicative of the method performance. We define “correctly estimated interactions” as cases where the effect of another individual on the movement of the focal individual was assessed as being significant when inter-individual interactions were indeed present (true positives) and the failure to detect the effect of another individual when inter-individual interactions were indeed absent in the simulated data (true negatives). For the scenarios in which the correlated movement is resulting from the effect of the physical environment (scenarios A, B, C), the estimate of inter-individual interaction should not be significant, while the estimate of environmental effects should be significant. The opposite is the case for scenario D, in which the correlated movement is resulting from individual interactions.

For both SSF-based approaches we fitted models with three different structures. First, the “Full” model included occurrence distribution of (SSF-OD) and the distance to (SSF-DIST) the non-focal individual as well as the physical environment as predictors. Second, the model “Individual” included occurrence distribution of (SSF-OD) and the distance to (SSF-DIST) the non-focal individual as a predictor to reflect the situation when no environmental data are available for the field researchers to include as a covariate (or, the available environmental data that can be included in the analyses are not relevant for the movement of the individuals). Third, in the model “Spatial+” we applied spatial+ to the “Individual” model. For this we first fitted the Generalised Additive Model (GAM) using OD of the non-focal individual or DIST to it as response variable and the thin plate spline of coordinates as independent predictor. We then used the residuals of this model as the corrected covariates and the spline of the coordinates in the SSF. We used 100 knots when fitting GAM because we did not want to limit the degree of non-linearity in the fitted relation. This is the practice suggested by the developers of spatial+ [12].

For Scenario C (barrier landscape), we addinonaly fitted a logistic regression for both SSFs approaches by using the proportion of the barriers as a predictor and the significance (“significant” or “not”) of the inferred interactions from models “Individual” and “Spatial+” as the binary response.

#### Scenarios A–C: observed correlated movement arises from interaction with environmental surroundings

We expect positive significant DI indices (type I error). Regarding both SSFs, we expect model “Individual” to wrongly detect interactions between individuals (type I error) and thus the coefficient to be positive and significant. If we apply Spatial+ (model “Spatial+ ”), we expect the coefficients to become non-significant for the SSFs. For model”Full” that includes also habitat quality as predictor in the analysis, we expect the two SSF-based approaches to correctly detect an attraction towards the physical environment as well as no interactions between the individuals. For scenario C we did not extract any physical environmental covariates (i.e. barriers as a covariate or distance to barriers) and thus did not fit the “Full” model.

#### Scenario D: observed correlated movement arises from direct interactions of individuals

We expect positive significant indices from DI. For the SSFs, we expect that all three models will correctly detect individual interactions, that is the coefficients will be positive and significant. We also expect the coefficents associated with environmental predictor to be non-significant for model “Full”.

#### Software

All analyses were perfromed in R—4.3.3 with the packages WildlifeDI—0.5.1, amt—0.2.2.0, survival—1.3–28.1 and mgcv—1.9–0.

## Results

### Scenario A: correlated movement caused by the resource gradient

#### Scenario A.a: moving next to each other

**Fig. 2:**
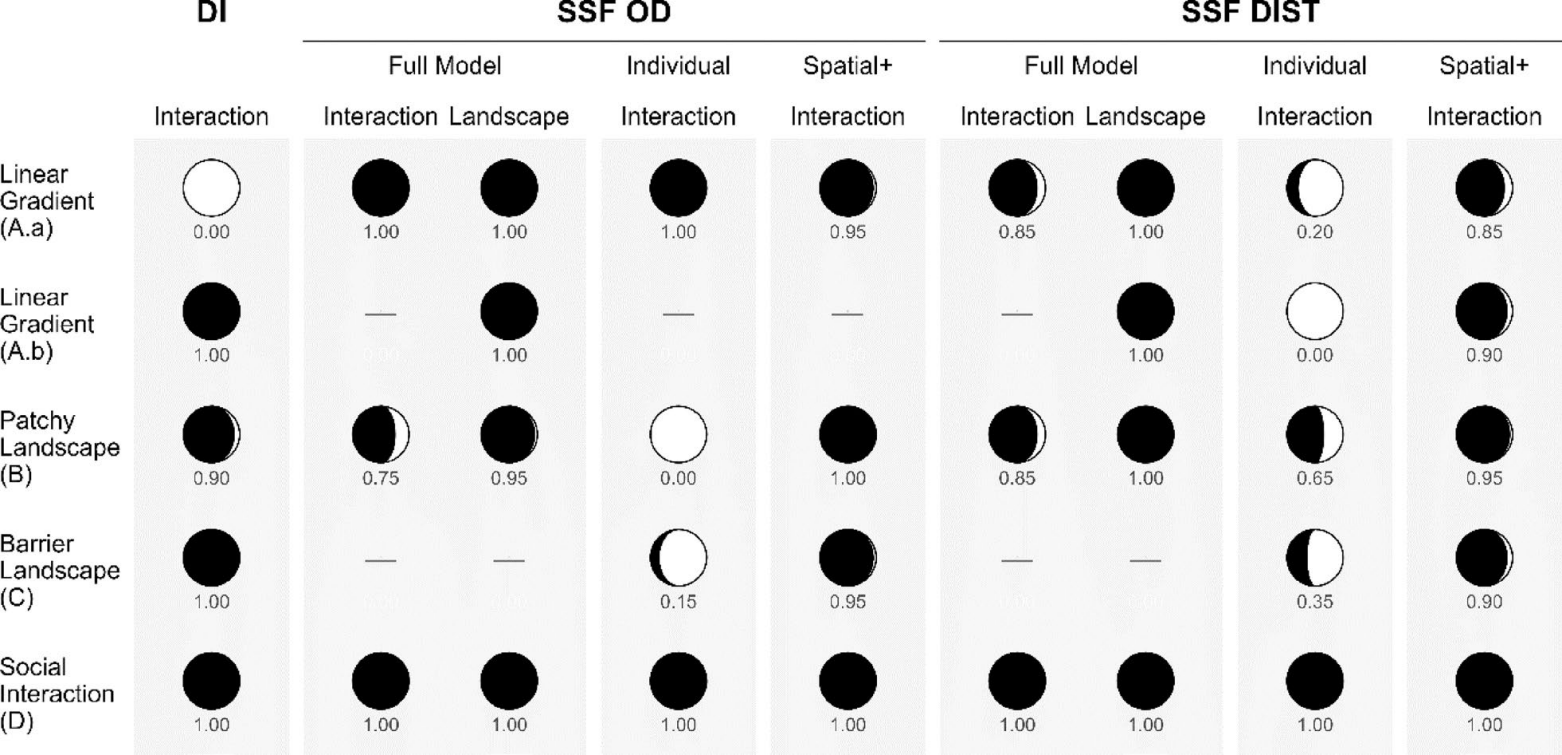
For each scenario, the proportion of cases in which DI, SSF-OD and SSF-DIST methods correctly detected whether the interactions are present are shown. For both SSFs three models were fitted; (1) a “Full” model with OD/DIST and landscape used as covariates, (2) an “Individual” model where only OD/DIST are used as covariate, (3) model “Spatial+” where Spatial+ was applied prior to fitting the model “Individual”. Columns with “Interaction” indicate the interaction between two individuals, while columns with “Landscape” indicate the attraction of the individual towards the landscape features. Cells with “-” indicate cases in which models could not be fitted (See text). The proportions for the scenario C were calculated setting the proportion of barriers in the landscape at 70%. For this scenario no full model was applied

**Fig. 3:**
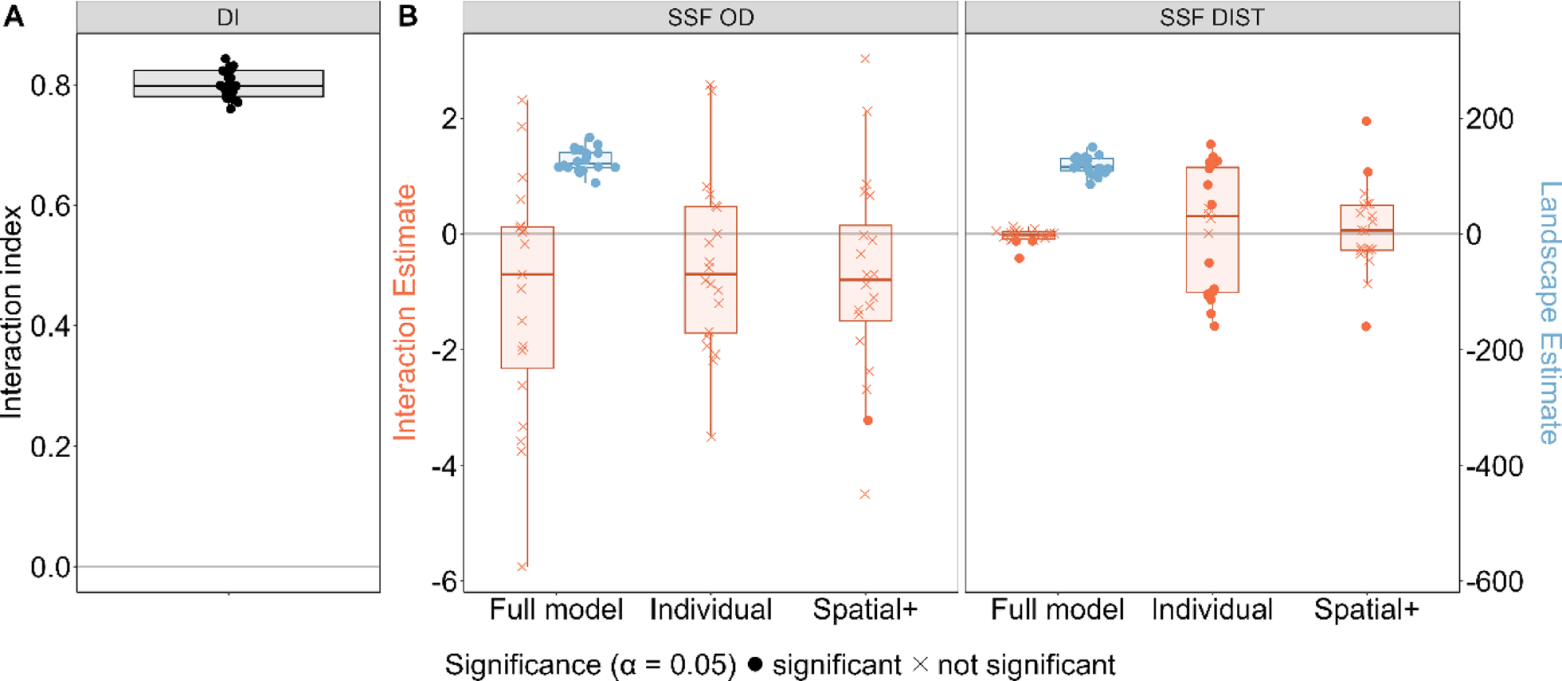
Performance of three statistical methods when applied to movement data generated under scenario **A.a**, correlated movement of two individuals moving next to each other caused by following a linear resource gradient. No inter-individual interaction but interactions with the physical landscape were simulated. Estimates were obtained by applying three methods to the simulated movement data: DI (panel **A**), SSF-OD & SSF-DIST (panel **B**). For both SSFs three models were fitted; (1) a “Full” model with OD/DIST and landscape used as covariates, (2) an “Individual” model with OD/DIST used as covariate, (3) Spatial+ was applied prior to fitting the “Individual” model. The estimates of the interactions between two individuals are shown in orange, while the estimates of the interaction between the focal individual and the landscape are shown in blue. The values that are significantly different from 0 (at *p* < 0.05) are shown with filled points and those that are not significantly different from 0 are shown with crosses

In the scenario A.a, where both individuals were moving next to each other along a habitat quality gradient, DI wrongly detected interactions between individuals (Fig. 2). For model “Individual” (DIST/OD as predictor) SSF-OD correctly detected no individual interactions, while SSF-DIST detected significant individual interactions (Fig. 3). Applying spatial+ to the SSF-OD had no strong impact on the estimates. For SSF-DIST, applying spatial+ prior to fitting the SSF resulted in removing the spurious effect of the individual interaction covariate (Figs. 2, 3). The “Full” model (DIST/OD and landscape as predictor) for both SSFs correctly revealed significant landscape estimates and non-significant interaction estimates. In this case SSF-OD returned high variation among the estimates of individual interactions, while under SSF-DIST such variation was much smaller.

#### Scenario A.b: moving from opposite locations towards the resource in the centre

**Fig. 4:**
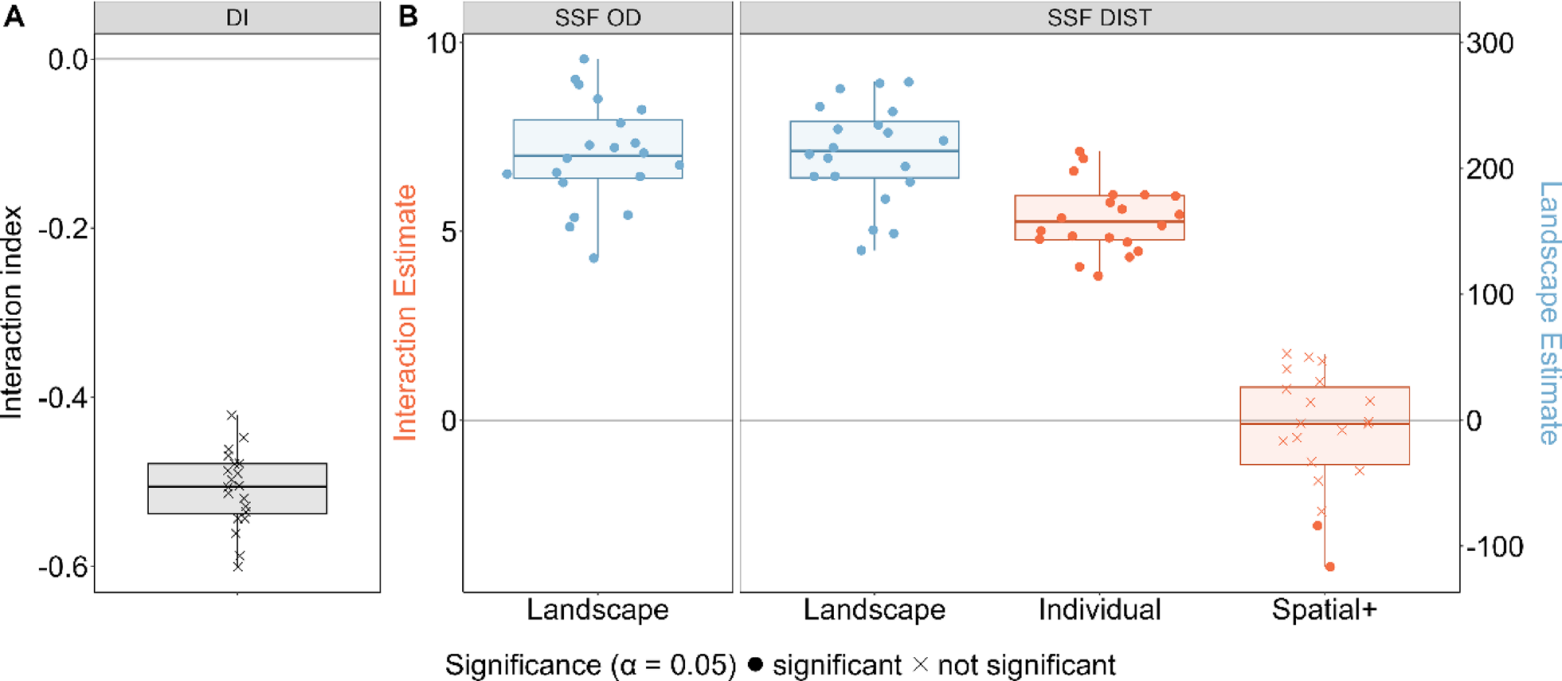
Performance of three statistical methods DI (panel **A**), SSF-OD & SSF-DIST (panel **B**) when applied to movement data generated under scenario **A.b**, correlated movement of two individuals moving from opposite locations towards the resource in the centre. No inter-individual interactions but interactions with the landscape were simulated. For both SSF-DIST & SSF-OD, “Full” model was fitted with landscape as the only predictor due to high collinearity between DIST/OD and landscape covariates. For SSF-OD model “Individual” and model “Spatial+ ” were not fitted due to the lack of overlapping movement paths. Annotations as in Fig. 3

DI correctly detected no interactions of individuals. For model “Individual” (DIST/OD as predictor), SSF-DIST detected individual interactions. Applying spatial+ to SSF-DIST removed the effect of the DIST covariate and thus the inter-individual interaction estimates became non-significant (Figs. 2, 4). Due to high correlation (Pearson correlation coefficient = − 0.97) between both predictors in model “Full” (DIST & landscape as predictor), we decided not to fit the full model. Therefore, in this scenario model “Full” was only fitted with the landscape covariate as predictor. For both SSFs model “Full” estimated the landscape effect as significant. For SSF-OD both models “Individual” and “Spatial+” were not shown, as SSF-OD is unable to accurately estimate the inter-individual interaction coefficient when the movement paths of the individuals do not overlap.

### Scenario B: correlated movement caused by the patchy landscape

**Fig. 5:**
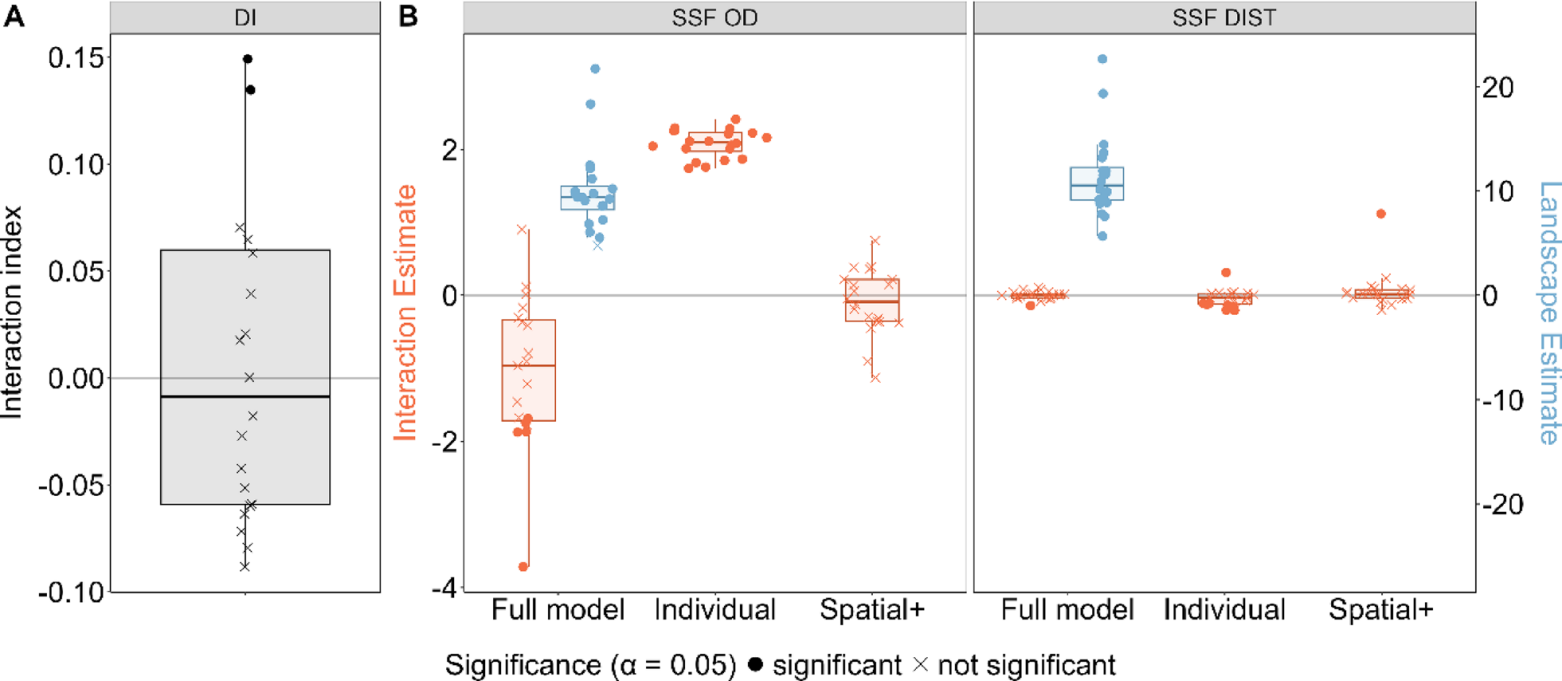
Performance of three statistical methods DI (panel **A**), SSF-OD & SSF-DIST (panel **B**) when applied to movement data generated under scenario B: correlated movement of two individuals moving in a patchy landscape, i.e. no social interaction between individuals but attraction of individuals to the physical landscape. Annotations as in Fig. 3

DI mostly correctly detected the absence of interactions between the two individuals (Fig. 2). For model “Individual” (DIST/OD as predictor) SSF-DIST, and especially, SSF-OD, erroneously detected significant inter-individual interactions. Applying spatial+ to both SSFs resulted in removing the spurious effect of the individual interaction covariate (Figs. 2,  5). The “Full” model (DIST/OD & landscape as predictor) fitted for both SSFs mostly correctly assessed landscape estimates as being significant and interaction estimates as non-significant.

### Scenario C: correlated movement caused by avoiding barriers

**Fig. 6:**
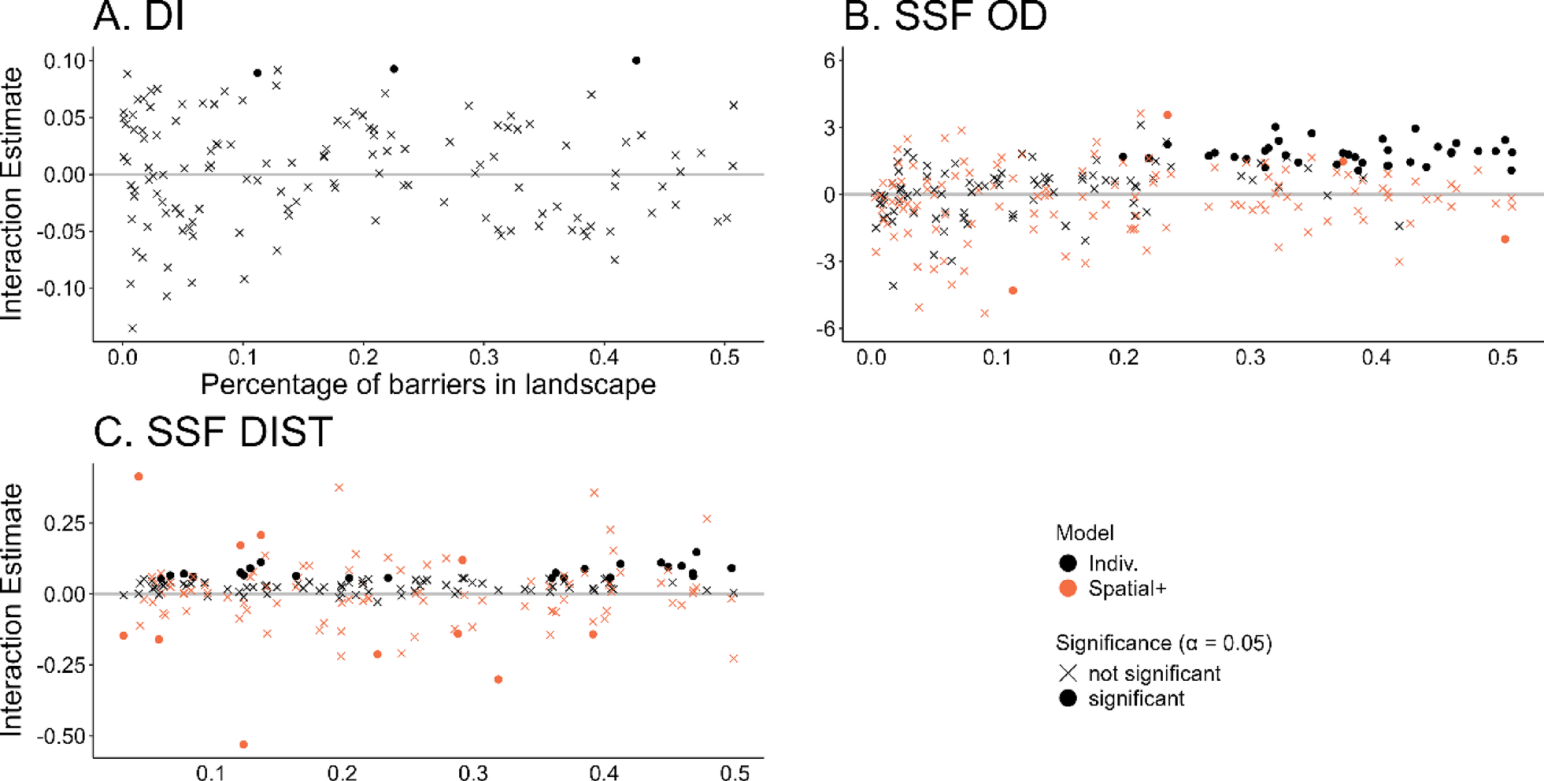
Performance of three statistical methods (**A**: DI, **B**: SSF-OD, **C**: SSF-DIST) when applied to movement data generated under scenario **C** correlated movement of two individuals due to their avoidance of barriers. The effect of the percentage of barriers in the landscape on the interaction estimate is shown in black colouring. For both SSFs two models were fitted; (1) OD/DIST were used as covariate, (2) spatial+ was applied prior to fitting the model “Individual” (orange colouring). The values that are significantly different from 0 (at *p* < 0.05) are shown with filled points and those that are not significantly different from 0 are shown with crosses

The percentage of barriers in the landscape did not affect the performance of DI (Fig. 6a). As the percentage of barriers increased in the landscape, SSF-OD and SSF-DIST falsely detected an increasing number of interactions between the individuals, where in fact there were none. This effect was mostly pronounced for SSF-OD as evidenced by the beta coefficient of a larger absolute magnitude: beta for SSF-OD = − 21.30 ± 3.90 and beta for SSF-DIST =− 5.51 ±− 0.96 (both significant at the *p *value < 0.001, Fig. 6b). Applying Spatial + effectively improved the performance of both SSF-OD and SSF-DIST, as fewer significant interaction estimates were detected under higher percentages of barriers in landscapes (Figs. 2 and 6) and the proportion of the barriers did not have an effect on the significance: beta for SSF-OD = 4.85 ± 3.42 and beta for SSF DIST = 0.61 ± 1.01 (both not significant at *p *value of 0.05). However, while applying Spatial+ with SSF-DIST some falsely significant estimates were detected, irrespective of the proportion of the barriers.

### Scenario D: correlated movement caused by individual interactions

**Fig. 7:**
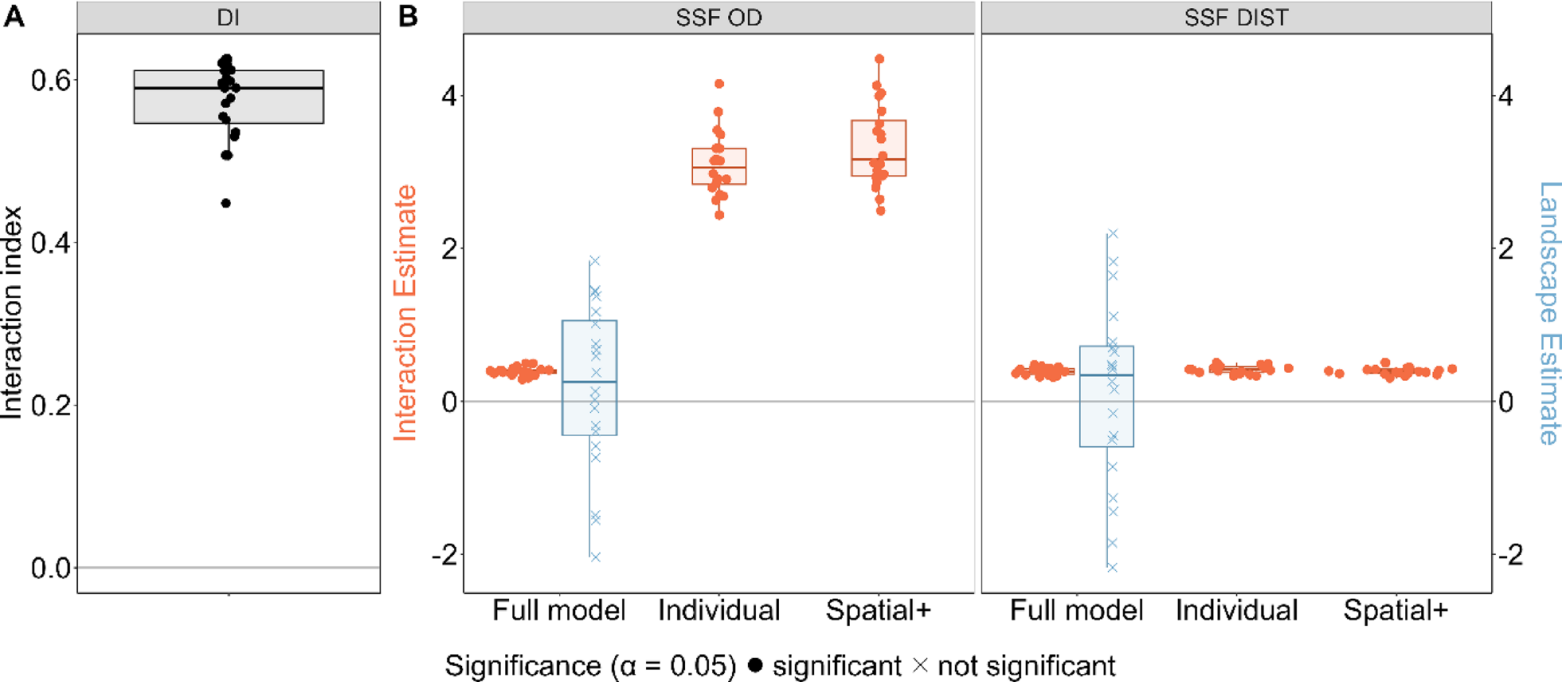
Performance of three statistical methods DI (panel **A**), SSF-OD & SSF-DIST (panel **B**) when applied to movement data generated under scenario D: correlated movement of two individuals due to their attraction to each other and not due to landscape effects. Annotations as in Fig. 3

All three methods correctly detected individual interactions, yielding significant positive estimates (Fig. 2, Fig. 7). Applying spatial+ did not change this outcome. Furthermore, both SSF-based approaches correctly detected significant interaction estimates and non- significant landscape estimates in the full model (Fig. 7).

## Discussion

Here we assessed how a heterogeneous environment affects the inference of inter-individual-interactions from movement data. One of the commonly used indices DI does not consider the physical environment at all and it did not perform that well, especially when correlation in movement tracks was driven by habitat. On the contrary, SSFs allow disentangling the effects of direct inter-individual interactions (i.e. social factors) from environmental effects (i.e. physical factors) on the movement of individuals. Including the physical environment as a predictor in SSFs increased the performance by decreasing the rate of false positives. However, fitting such models with both the effect of another individual and of the landscape on the movement of the focal individual was not possible when they were highly collinear. In contrast, by applying the novel Spatial+ method to SSFs that only included the movement of other individuals as covariate we could demonstrate consistent increase in method performance.

In our scenarios A.a and A.b in which the simulated animals do not interact with each other directly but move persistently along a linear habitat gradient, we have shown that neglecting the physical environment results in falsely detecting interactions between the two individuals by SSF-DIST. Including the landscape as a covariate in the step selection function reduced the rates of falsely detected inter-individual-interactions. However, including both the social and physical environment as predictors in the model is not always possible, since they can become highly collinear. For instance, we simulated such a collinearity in our scenario A.b in which two individuals move from opposite locations towards the same resource in the middle, such that the value of the habitat quality increases while the distance to the other individual decreases. In such cases, revealing the true effect will be difficult or even impossible from a statistical perspective and the focus should be on gaining understanding of how animals interact with their environment prior to analysing inter-individual interactions. Especially when interested in commuting, dispersal or migration movements, persistent movement along linear habitat gradients/features can occur regularly [7, 20, 25, 38]. For instance, for temperate seasonal migrating animals such as woodland caribou or elk, the movement is strongly attributed to seasonal environmental conditions, resulting in following the vegetative growth during spring [3, 20]. Other examples of such movement patterns are found in savannas where different animals are attracted to the same food/water resources, thereby forming big aggregations of different species moving together [19]. Such large-scale movements are surely driven by both interactions among individuals as well as by their attraction to the landscape. In real systems, thus, the situation may be not as “black-and-white” as depicted by our artificial scenarios that were designed to capture extreme situations. Further, real landscapes are often heterogeneous. Heterogeneity was on purpose omitted in our “extreme” scenarios A.a and A.b, while scenario B sought to mimic such heterogeneous landscapes. Thus, the collinearity in the data collected in real landscapes is likely to be weaker and hopefully less of an issue.

In the heterogeneous landscape of scenario B, we also found similar responses as in the two first scenarios A.a and A.b: When the landscape was neglected, SSF-DIST and especially SSF-OD falsely detected a high rate of interactions between the individuals. Including the physical environment as a covariate reduced the rate of false positives for the SSFs. The movements modelled in such a patchy landscape reflect movements during foraging behaviour of animals, which are expressed by many animals on a daily basis, with many species being attracted to the same resource [1, 38]. For example, social zebrafish use a combination of the physical and social environment to increase their foraging efficiency [26]. An aggregation of zebrafishes at a food resource could therefore primarily result from the attraction towards the same resource. This reinforces the difficulty to distinguish whether the physical or the biotic environment affected the movement, since the physical environment can act as attractor or facilitator.

While it is acknowledged that animal space use is affected by both inter-individual-interactions as well as the physical environment, it is often not addressed by leaving the physical descriptors out of the analyses, especially when inter-individual interactions are the point of interest. Yet, neglecting the physical environment could result in a so-called spatial cofounding, meaning the covariate of interest (here: coefficient of inter-individual interaction) is spatially dependent, thus resulting in unreliable estimates [2, 21, 52]. Omitting these unobserved spatial variables results in unexplained spatial variation, which then leads to a correlation in the residuals. In such cases when no environmental data is available, applying Spatial+ in addition to the step selection function allows reducing spatial cofounding in animal movement and leads to an improved inference of inter-individual-interactions, according to our findings (A.a, A.b & C).

Further, an extension of SSFs, called iSSFs, can be used [4, 14]. In addition to estimating the animals´ selection of their environment, they also allow estimating the effects of those environments on step lengths and turning angles, and therefore inferring the movement characteristics of the animals. Although this method does not resolve the issue of highly collinear variables, it might help to identify different movement modes of animals. For example, in a “foraging state” the animal might be attracted by high food quality patches and therefore the movement parameters are characterized by small step lengths and more uniform turning angles. While in a “mate-searching” or “hunting” state the movement parameters are characterized by longer step lengths and high directional persistency. Moreover, animals switch between different movement states over time. Such switches in behaviours could be detected by applying Hidden Markov model—SSF [30, 42] which is a new approach that contributes to improve inference of inter-individual interactions, as it allows detecting changes in behavioural states due to their environment. This allows then to split the movement trajectory into meaningful chunks depending on the behavioural states of the individuals.

In scenario C we revealed the emerging bias of correlated movements due to increasing habitat fragmentation. For both SSFs, with increasing proportion of obstacles (non-favourable elements) in the landscape, an increase in falsely detected social interactions was observed. Applying Spatial+ was again successful in removing this spatial cofounding when applied to SSF-OD, reducing the rates of falsely detected social interactions. This finding is of high importance when analysing individual movement in fragmented landscapes. As the landscape fragmentation increases, hostile matrix acting as a barrier becomes larger and thus the space use of animals becomes restricted to smaller areas [13]. This then results in a higher overlap in movement paths of individuals, causing higher correlation in their movement trajectories that is not necessarily due to the direct interactions between individuals. Furthermore, linear features in the landscape used by many animals [11] can cause such correlations in animal movement. For example, corridors in highly fragmented landscapes act as “drift fences”, intercepting and redirecting the movement of many animals [24], thus potentially increasing the detection of interactions between individuals that might only be due to them using the same space. Wildlife crossings such as green bridges over highways, used by wolves, deer and wild boar [41], might also increase the potential of spuriously detecting inter-individual interactions. Another example of animals using linear structures as guidance for navigation are golden eagles who use the Rocky Mountain range to migrate to the north [7]. Thus, including such landscape structure as a covariate in the analyses is of high importance to account for this bias. However, one must be careful when interpreting the estimates of an SSF. In case an animal uses an environmental structure as guidance and not as a resource, for example wild boar moving along forest patches [50] but never entering that landscape structure, the SSFs will detect an avoidance of that structure [51].

In scenario D we have shown that including physical environmental data as predictors in the model does not worsen the inference of inter-individual interactions. We advise that field ecologists should be more cautious about false positives, as it seems that false negatives are less likely to be of an issue than false positives in a heterogeneous landscape, at least in the investigated scenarios.

The addressed two SSF methods [43, 45], show promising results in dealing with confounding factors when assessing inter-individual interactions of moving individuals. We show that especially when the physical environment strongly affects the animal movement, including physical environmental data or applying Spatial+ [12] is essential to improve inference of inter-individual interactions and avoid detection of spurious effects. Yet, rarely all the covariates that could strongly influence the movement of the tracked animals are considered in the analysis. For example, such missing covariates can be unobserved individuals that interact with the tracked individuals, which is a big issue that practitioners are facing (see [18] for possible implications of such untracked individuals). Similarly, the presence of predators in the landscape that cause the tracked individuals to avoid specific areas without the predators to directly interact with them (landscape of fear) may cause spatial structure in movement trajectories. Since knowing all relevant environmental covariates is virtually impossible, we strongly advocate for using Spatial+ when the aim is to infer inter-individual interactions from movement trajectories. But, of course, no method will be able to replace understanding of the species biology and thus prior knowledge of the resource use by the study species and detailed understanding of its spatial ecology is the key. We suggest that, when interested in inter-individual interactions, collecting movement data should, whenever possible, be accompanied by the collection of environmental data relevant to the study species.

## Supplementary Information

Additional file1 (DOCX 67 kb)

## Data Availability

The codes used to simulate the data as well as the codes for analyses of the simulated data will be made available on GitHub upon acceptance of the manuscript.

## Abbreviations

SSF: Step selection function
OD: Occurrence distribution
DIST: Distance
iSSF: Integrated step selection function
HMM: Hidden Markov model
DI: Dynamic interaction
ABM: Agent based model
ODD: Overview, design, concepts, details

## References

[1] Abrahms B, Aikens EO, Armstrong JB, Deacy WW, Kauffman MJ, Merkle JA. Emerging perspectives on resource tracking and animal movement ecology. Trends Ecol Evol. 2021;36(4):308–20. 10.1016/j.tree.2020.10.018. 33229137 10.1016/j.tree.2020.10.018

[2] Arce Guillen R, Lindgren F, Muff S, Glass TW, Breed GA, Schlägel UE. Accounting for unobserved spatial variation in step selection analyses of animal movement via spatial random effects. Methods Ecol Evol. 2023;14(10):2639–53. 10.1111/2041-210X.14208.

[3] Avgar T, Mosser A, Brown GS, Fryxell JM. Environmental and individual drivers of animal movement patterns across a wide geographical gradient. J Anim Ecol. 2013;82(1):96–106. 10.1111/j.1365-2656.2012.02035.x. 23020517 10.1111/j.1365-2656.2012.02035.x

[4] Avgar T, Potts JR, Lewis MA, Boyce MS. Integrated step selection analysis: bridging the gap between resource selection and animal movement. Methods Ecol Evol. 2016;7(5):619–30. 10.1111/2041-210X.12528.

[5] Banks SC, Piggott MP, Stow AJ, Taylor AC. Sex and sociality in a disconnected world: a review of the impacts of habitat fragmentation on animal social interactions. Can J Zool. 2007;85(10):1065–79. 10.1139/Z07-094.

[6] Bastille-Rousseau G, Potts JR, Yackulic CB, Frair JL, Ellington E, Hance Blake Stephen. Flexible characterization of animal movement pattern using net squared displacement and a latent state model. Mov Ecol. 2016;4:15. 10.1186/s40462-016-0080-y. 27252856 10.1186/s40462-016-0080-y PMC4888472

[7] Bedrosian BE, Domenech R, Shreading A, Hayes MM, Booms TL, Barger CR. Migration corridors of adult Golden Eagles originating in northwestern North America. PLoS ONE. 2018;13(11): e0205204. 10.1371/journal.pone.0205204. 30462652 10.1371/journal.pone.0205204 PMC6248900

[8] Benhamou S, Valeix M, Chamaillé-Jammes S, Macdonald DW, Loveridge AJ. Movement-based analysis of interactions in African lions. Anim Behav. 2014;90:171–80. 10.1016/j.anbehav.2014.01.030.

[9] Calabrese JM, Fleming CH, Fagan WF, Rimmler M, Kaczensky P, Bewick S, et al. Disentangling social interactions and environmental drivers in multi-individual wildlife tracking data. Philos Transe Roy Soc Lond Ser B Biol Sci. 2018. 10.1098/rstb.2017.0007. 10.1098/rstb.2017.0007 PMC5882977 29581392

[10] Cote J, Clobert J. Social information and emigration: lessons from immigrants. Ecol Lett. 2007;10(5):411–7. 10.1111/j.1461-0248.2007.01032.x. 17498140 10.1111/j.1461-0248.2007.01032.x

[11] Dickie M, McNay SR, Sutherland GD, Cody M, Avgar T. Corridors or risk? Movement along, and use of, linear features varies predictably among large mammal predator and prey species. J Anim Ecol. 2020;89(2):623–34. 10.1111/1365-2656.13130. 31648375 10.1111/1365-2656.13130 PMC7028095

[12] Dupont E, Wood SN, Augustin NH. Spatial+: a novel approach to spatial confounding. Biometrics. 2022;78(4):1279–90. 10.1111/biom.13656. 35258102 10.1111/biom.13656 PMC10084199

[13] Eycott AE, Stewart GB, Buyung-Ali LM, Bowler DE, Watts K, Pullin AS. A meta-analysis on the impact of different matrix structures on species movement rates. Landsc Ecol. 2012;27(9):1263–78. 10.1007/s10980-012-9781-9.

[14] Fieberg J, Signer J, Smith B, Avgar T. A “How to” guide for interpreting parameters in habitat-selection analyses. J Anim Ecol. 2021;90(5):1027–43. 10.1111/1365-2656.13441. 33583036 10.1111/1365-2656.13441 PMC8251592

[15] Fleming CH, Fagan WF, Mueller T, Olson KA, Leimgruber P, Calabrese JM. Rigorous home range estimation with movement data: a new autocorrelated kernel density estimator. Ecology. 2015;96(5):1182–8. 10.1890/14-2010.1. 26236833 10.1890/14-2010.1

[16] Forester JD, Im HK, Rathouz PJ. Accounting for animal movement in estimation of resource selection functions: sampling and data analysis. Ecology. 2009;90(12):3554–65. 10.1890/08-0874.1. 20120822 10.1890/08-0874.1

[17] Fortin D, Beyer HL, Boyce MS, Smith DW, Duchesne T, Mao JS. Wolves influence elk movements: behavior shapes a trophic cascade in Yellowstone national park. Ecology. 2005;86(5):1320–30. 10.1890/04-0953.

[18] Fronville T, Blaum N, Kramer-Schadt S, Schlägel U, Radchuk V. Performance of five statistical methods to infer interactions among moving individuals in a predator–prey system. Methods Ecol Evol. 2024;15(6):1097–112. 10.1111/2041-210X.14323.

[19] Fryxell JM, Sinclair AR. Causes and consequences of migration by large herbivores. Trends Ecol Evol. 1988;3(9):237–41. 10.1016/0169-5347(88)90166-8. 21227239 10.1016/0169-5347(88)90166-8

[20] Fryxell JM, Avgar T. Animal migration: catching the wave. Nature. 2012;490(7419):182–3. 10.1038/490182a. 23060184 10.1038/490182a

[21] Gilbert B, Datta A, Casey JA, Ogburn EL. A causal inference framework for spatial confounding 2021.

[22] Grimm V, Berger U, Bastiansen F, Eliassen S, Ginot V, Giske J, et al. A standard protocol for describing individual-based and agent-based models. Ecol Model. 2006;198(1–2):115–26. 10.1016/j.ecolmodel.2006.04.023.

[23] Grimm V, Railsback SF, Vincenot CE, Berger U, Gallagher C, DeAngelis DL, et al. The ODD protocol for describing agent-based and other simulation models: a second update to improve clarity, replication, and structural realism. JASSS. 2020;23(2):7. 10.18564/jasss.4259.33204215

[24] Haddad NM, Bowne DR, Cunningham A, Danielson BJ, Levey DJ, Sargent S, Spira T. Corridor use by diverse taxa. Ecology. 2003;84(3):609–15. 10.1890/0012-9658(2003)084[0609:CUBDT]2.0.CO;2.

[25] Hahn TP, Cornelius JM, Sewall KB, Kelsey TR, Hau M, Perfito N. Environmental regulation of annual schedules in opportunistically-breeding songbirds: adaptive specializations or variations on a theme of white-crowned sparrow? General Compar Endocrinol. 2008;157(3):217–26. 10.1016/j.ygcen.2008.05.007. 10.1016/j.ygcen.2008.05.007 18602554

[26] Harpaz R, Schneidman E. Social interactions drive efficient foraging and income equality in groups of fish. Elife. 2020. 10.7554/eLife.56196. 32838839 10.7554/eLife.56196 PMC7492088

[27] He P, Maldonado-Chaparro AA, Farine DR. The role of habitat configuration in shaping social structure: a gap in studies of animal social complexity. Behav Ecol Sociobiol. 2019. 10.1007/s00265-018-2602-7.

[28] Herbert-Read JE, Rosén E, Szorkovszky A, Ioannou CC, Rogell B, Perna A, et al. How predation shapes the social interaction rules of shoaling fish. Proc Biol Sci. 2017. 10.1098/rspb.2017.1126. 28855361 10.1098/rspb.2017.1126 PMC5577484

[29] Isbell LA. Contest and scramble competition: patterns of female aggression and ranging behavior among primates. Behav Ecol. 1991;2(2):143–55. 10.1093/beheco/2.2.143.

[30] Klappstein NJ, Michelot T, Fieberg J, Pedersen EJ, Flemming M, Joanna. Step selection functions with non-linear and random effects. Methods Ecol Evol. 2024;15(8):1332–46. 10.1111/2041-210X.14367.

[31] Laundre JW, Hernandez L, Ripple WJ. The landscape of fear: ecological implications of being afraid~!2009-09-09~!2009-11-16~!2010-02-02~! TOECOLJ. 2010;3(3):1–7. 10.2174/1874213001003030001.

[32] Long JA, Nelson TA. Measuring dynamic interaction in movement data. Trans GIS. 2013;17(1):62–77. 10.1111/j.1467-9671.2012.01353.x.

[33] Long JA, Nelson TA, Webb SL, Gee KL. A critical examination of indices of dynamic interaction for wildlife telemetry studies. J Anim Ecol. 2014;83(5):1216–33. 10.1111/1365-2656.12198. 24428545 10.1111/1365-2656.12198

[34] Marescot L, Franz M, Benhaiem S, Hofer H, Scherer C, East ML, Kramer-Schadt S. “Keeping the kids at home” can limit the persistence of contagious pathogens in social animals. J Anim Ecol. 2021;90(11):2523–35. 10.1111/1365-2656.13555. 34118063 10.1111/1365-2656.13555

[35] Nathan R, Getz WM, Revilla E, Holyoak M, Kadmon R, Saltz D, Smouse PE. A movement ecology paradigm for unifying organismal movement research. Proc Natl Acad Sci USA. 2008;105(49):19052–9. 10.1073/pnas.0800375105. 19060196 10.1073/pnas.0800375105 PMC2614714

[36] Nathan R, Monk CT, Arlinghaus R, Adam T, Alós J, Assaf M, et al. Big-data approaches lead to an increased understanding of the ecology of animal movement. Science (New York NY). 2022;375(eabg6582):1780. 10.1126/science.abg1780. 10.1126/science.abg1780 35175823

[37] Niu Mu, Blackwell PG, Skarin A. Modeling interdependent animal movement in continuous time. Biometrics. 2016;72(2):315–24. 10.1111/biom.12454. 26812666 10.1111/biom.12454

[38] Owen-Smith N, Hopcraft G, Morrison T, Chamaillé-Jammes S, Hetem R, Bennitt E, van Langevelde F. Movement ecology of large herbivores in African savannas: current knowledge and gaps. Mammal Rev. 2020;50(3):252–66. 10.1111/mam.12193.

[39] Perlin K. An image synthesizer. SIGGRAPH Comput Graph. 1985;19(3):287–96. 10.1145/325165.325247.

[40] Pinter-Wollman N, Fiore SM, Theraulaz G. The impact of architecture on collective behaviour. Nat Ecol Evol. 2017;1(5):111. 10.1038/s41559-017-0111. 28812703 10.1038/s41559-017-0111

[41] Plaschke M, Bhardwaj M, König HJ, Wenz E, Dobiáš K, Ford AT. Green bridges in a re-colonizing landscape: wolves (*Canis lupus*) in Brandenburg, Germany. Conserv Sci Pract. 2021;3(3):364. 10.1111/csp2.364.

[42] Pohle J, Signer J, Eccard JA, Dammhahn M, Schlägel UE. How to account for behavioral states in step-selection analysis: a model comparison. PeerJ. 2024;12: e16509. 10.7717/peerj.16509. 38426131 10.7717/peerj.16509 PMC10903358

[43] Roeleke M, Schlaegel UE, Gallagher C, Pufelski J, Blohm T, Nathan R, et al. Insectivorous bats form mobile sensory networks to optimize prey localization: The case of the common noctule bat. Proc Natl Acad Sci USA. 2022;119(33): e2203663119. 10.1073/pnas.2203663119. 35939677 10.1073/pnas.2203663119 PMC9388074

[44] Schlaegel UE, Grimm V, Blaum N, Colangeli P, Dammhahn M, Eccard JA, et al. Movement-mediated community assembly and coexistence. Biol Rev Camb Philos Soc. 2020;95(4):1073–96. 10.1111/brv.12600. 32627362 10.1111/brv.12600

[45] Schlaegel UE, Signer J, Herde A, Eden S, Jeltsch F, Eccard JA, Dammhahn M. Estimating interactions between individuals from concurrent animal movements. Methods Ecol Evol. 2019;10(8):1234–45. 10.1111/2041-210X.13235.

[46] Signer J, Fieberg J, Reineking B, Schlägel U, Smith B, Balkenhol N, Avgar T. Simulating animal space use from fitted integrated Step-Selection Functions (iSSF ). Methods Ecol Evol. 2024;15(1):43–50. 10.1111/2041-210X.14263.

[47] Spiegel O, Leu ST, Sih A, Bull CM. Socially interacting or indifferent neighbours? Randomization of movement paths to tease apart social preference and spatial constraints. Methods Ecol Evol. 2016;7(8):971–9. 10.1111/2041-210X.12553.

[48] Strandburg-Peshkin A, Farine DR, Crofoot MC, Couzin ID. Habitat and social factors shape individual decisions and emergent group structure during baboon collective movement. Elife. 2017. 10.7554/eLife.19505. 28139196 10.7554/eLife.19505 PMC5283833

[49] Tang W, Bennett DA. Agent-based modeling of animal movement: a review. Geogr Compass. 2010;4(7):682–700. 10.1111/j.1749-8198.2010.00337.x.

[50] Thurfjell H, Ball JP, Åhlén P-A, Kornacher P, Dettki H, Sjöberg K. Habitat use and spatial patterns of wild boar *Sus scrofa* (L.) agricultural fields and edges. Eur J Wildl Res. 2009;55(5):517–23. 10.1007/s10344-009-0268-1.

[51] Thurfjell H, Ciuti S, Boyce MS. Applications of step-selection functions in ecology and conservation. Mov Ecol. 2014;2(1):4. 10.1186/2051-3933-2-4. 25520815 10.1186/2051-3933-2-4 PMC4267544

[52] Urdangarin A, Goicoa T, Ugarte MD. Evaluating recent methods to overcome spatial confounding. Rev Mat Complut. 2023;36(2):333–60. 10.1007/s13163-022-00449-8.

[53] Valeix M, Loveridge AJ, Davidson Z, Madzikanda H, Fritz H, Macdonald DW. How key habitat features influence large terrestrial carnivore movements: waterholes and African lions in a semi-arid savanna of north-western Zimbabwe. Landsc Ecol. 2010;25(3):337–51. 10.1007/s10980-009-9425-x.

[54] van Schaik CP. Why are diurnal primates living in groups? Behaviour. 1983;87(1–2):120–44. 10.1163/156853983X00147.

