# Supplementary material for: Considering landscape heterogeneity improves the inference of inter-individual interactions from movement data
Source: Mov Ecol. 2025 Jun 12;13:41. doi: 10.1186/s40462-025-00567-0 (PMC12160437; doi:10.1186/s40462-025-00567-0)
Supplement: Supplementary file 1 — Additional file1 (DOCX 67 kb) [file 40462_2025_567_MOESM1_ESM.docx]

**Appendix**

**Supplementary Methods**

***Dynamic interaction index (DI)***

The DI index measures the cohesiveness of simultaneous movement vectors with respect to two independent components of movement: distance (also called displacement) and direction (DI; Long and Nelson 2013). DI consists of two measures: it calculates cohesiveness in displacement and direction between two individuals for a defined time period, referred to as di, which is then used to calculate DI.

$di= \left( 1-\frac{\left| d_{t}^{\alpha}-d_{t}^{\beta} \right|}{d_{t}^{\alpha}+d_{t}^{\beta}} \right)\cdot\cos(\theta_{t}^{\alpha}-\theta_{t}^{\beta}) DI= \frac{1}{n-1} \sum_{t=1}^{n-1} di$

Here, $d$ is the displacement and $\theta$ the direction of a vector at a time period $t$ for both individuals $\alpha$ and $\beta$ within the trajectory of *n* number of fixes. The localized *di* is calculated by incorporating displacement and direction of both individuals for each time period. Thus, the localized *di* can distinguish between cohesiveness in distance ${di}_{d}$ (Measures dynamic interaction in distance: called displacement) and cohesiveness in movement direction${di}_{\theta}$ (Measures dynamic interaction in movement direction: called Azimuth). Values for DI range from -1 and 1 where negative values correspond to repulsive movement paths (opposite direction) and positive values indicate cohesive movement paths (in same direction). Values around 0 indicate neutral movement.

The DI method is separable into components measuring dynamic interaction in movement direction (azimuth) and movement distance (displacement), termed DIθ and DId respectively.

***SSF-based approaches***

The SSFs compare the observed steps of a focal individual to randomly sampled possible steps in terms of certain covariates, which allows estimating a selection strength for those covariates. In this particular case where the interactions with other individuals are in focus, as covariate we use the values of the occurrence distribution of other individuals. The random steps for the focal individual are obtained using the distribution of turning angles (usually following a von Mises distribution) and step length distribution (gamma or exponential distribution) of the actual individual´s movement path. If the estimates of selection strength are positive, we interpret this as an attraction of a focal individual to the other one; if selection strength is negative, this means avoidance. Estimates around zero suggest neutral behaviour of the focal individual towards the other one. Compared to DI, which can only be applied to movement data of two individuals at a time, the SSF-based approaches can be used to analyse the response of a focal individual towards multiple individuals. Further, multiple covariates can be considered, allowing to simultaneously analyse the effect of other individuals and landscape variables on the movement of a focal individual. Importantly, the coefficients are not bound between -1 and +1, but their scale depends on the scale of the covariates used. This means that care should be taken when comparing coefficients obtained for different covariates and that the coefficients should only be interpreted with their corresponding p-value.

The SSF-based approach by (Schlaegel et al. 2019) uses as a covariate dynamic occurrence distributions of other individuals (Fleming et al. 2015) and is referred to as SSF-OD approach in this study. Animal movement was typically sampled at rather low temporal resolutions in the past and thus the real path was not known. To deal with this, SSF-OD applies kriging to collected movement paths (e.g. arising from telemetry and GPS data, Fleming et al. 2015), which allows estimating the probability that an animal occurred within an area at a given time period. This probability is referred to as occurrence distribution (henceforth “OD”). Kriging yields a dynamic spatial map of the animal’s occurrence within given time periods where values close to one indicate locations that the individual has most likely visited and values close to zero indicate locations where the animal most likely did not occur. SSF are then applied to assess how such occurrence distribution of one individual affects the movement of another (focal) individual.

The second SSF-based approach we use is a modification of SSF-OD that, instead of the occurrence distribution, uses distances between individuals as covariates in SSFs (Roeleke et al. 2022). We refer to this approach as SSF-DIST. SSF-DIST uses the distance of a focal animal to other animals at each given time period. SSFs are then applied to assess whether the focal individual moves closer to or further away from the other individuals compared to random locations. In this case, larger distances between two individuals result in positive estimates of selection strength, meaning that such positive values reflect avoidance. Shorter distances, on contrary, result in negative estimates of selection strength, which are interpreted as attraction. In our study, for a better comparison of the results obtained with both SSF-methods, we multiplied the selection strength estimates obtained with SSF-DIST by -1, so that positive values indicate attraction and negative values indicate repulsion, exactly like in SSF-OD approach.

| Method | Reference | Description | Interpretation |
| --- | --- | --- | --- |
| Dynamic interaction index (DI) | Long & Nelson (2013) | Cohesion in the movement segments of two animals, with respect to distance and direction. | DI ~ + 1 Cohesive movement DI ~ - 1 Repulsive movement DI ~ 0 Neutral |
| Step-selection function approach based on occurrence distributions (SSF-OD) | Schlaegel et al. (2019) | Step-selection functions applied to occurrence distributions of individuals obtained through kriging of their movement path | SSF ~ + 1 Attraction SSF ~ - 1 Avoidance SSF ~ 0 Neutral |
| Step-selection function approach based on distances between individuals (SSF-DIST) | Roeleke et al. (2022) | Step-selection functions applied to distances between two individuals | SSF ~ + 1 Attraction SSF ~ - 1 Avoidance SSF ~ 0 Neutral |

***Table S1****: Overview of the three methods used in this study to assess interactions from movement data.*

# ODD Protocol

The model description follows the ODD protocol for describing agent-based models by Grimm et al. (2006) and updated (Grimm et al. 2020). The landscape generating simulation was implemented in Python version 3.10 and movement simulation were done in R version 4.1.0 with the user interface R-Studio version 1.4.1717.

1. Purpose and patterns

The purpose of this simulation model is to reproduce realistic movement of animals interacting either with their physical environment or with other moving individuals. The movement data simulated with this model is then used to assess how well the statistical methods can infer whether the animal is interacting with the physical environment or other animals.

1. Entities, state variables and scales

The simulation contains two types of moving agents. Either the agents only interact with their physical environment or with other moving individuals. State variables can be seen in Table 1.

| **Agent** | **Variables** | **Details** | **Unit** |
| --- | --- | --- | --- |
| Moving Individuals | Location | Current X,Y Coordinates of agents in gridded landscape | [X,Y] |
|  | Turning angle | Difference in headings at time step t and t-1 | rad |
|  | Step length | Distance of the next movement step derived from Gamma distribution | Euclidian distance |
|  | Movement boundaries | Available steps characterized by their turning angle and step length | [X,Y] |
|  | Available steps | Number of available steps | / |
|  | Selection coefficient | Selection strength towards the environment | / |
|  | Redistribution kernel | Probability of moving to new position defined by their movement boundaries and their environment | / |

**Table 1**: State variables of the two different mobile agents within the gridded landscape

The agents interacting with the physical environment were designed to mimic animals searching for resources or shelter. They are attracted towards the physical environment with a given attraction strength. The agents interacting with other individuals do not interact with the physical environment and are designed to mimic animals forming herds and moving in groups. Both agents are characterized by their coordinates, turning angle, step length, movement boundaries, available steps, selection coefficient and redistribution kernel.

The grid-based environment has closed borders and consists of 1000 x 1000 cells. Two different landscapes were generated. In the first environment the cells reflect habitat suitability. The values of habitat suitability range from zero to one. These cells can be arranged in such a way that the suitability of the cells was increasing linearly across the simulated area to the other (gradient) or the grid cells were assigned with a value generated with the Perlin noise function (Perlin 1985). The Perlin noise is used to generate not completely random values (Perlin 1985) and is helpful to create procedurally generated landscapes. This produces patches of grid cells with values similar to each other, which gives the appearance of more naturally clustered patchy landscapes compared to full randomness. In the second environment, the Perlin noise function is also used to generate barriers, that block the path of the individuals. In this case, the barrier cells receive the value 0 which is not selected for by the individuals (mimicking avoidance). The other cells are given a value of 1 to which the agents neither express attraction nor avoidance. Thus, they only move according to their movement characteristics and are repulsed by the barriers.

Process overview and scheduling

At each time step, two agents move to a new position within the landscape defined by their movement characteristics (turning angle and step length) and the attributes/values of the surrounding environment cells. Before each step, 1000 available steps are generated given a turning angle distribution and step length distribution. We used a von Mises distribution to generate the turning angles, and a gamma distribution for the step length. A selection-free movement kernel can be calculated given the movement characteristics. At each end points of these available steps the environmental attributes were then extracted. Given a selection coefficient (can induce preference or avoidance of the landscape) and the environmental attributes, a movement-free habitat selection function can be calculated. Given the selection-free movement kernel and the movement-free habitat selection function, a redistribution kernel (Avgar et al. 2016, Fieberg et al. 2021) can be calculated. The redistribution kernel is then the determining factor for the selection of the new step from the available steps. Available steps outside of the designated landscape area were omitted, so that the agents would not select for them and thus would not leave the designated landscape area.

1. Design Concepts

*Basic principles*. Movement patterns are strongly shaped by individual decisions which, in return, are influenced by their environment with its dynamic risks and rewards but also by their complex internal state (Nathan et al. 2008) and their movement constraints. The current version of the simulation is based on the assumption, that the presence of other con- or heterospecific agents around the individual´s location as well as the physical environment influence its decision about the next movement (Cote and Clobert 2007; Clobert et al. 2009).

*Emergence*. Since the purpose of the model is to simulate movement data, on which statistical methods will be applied, the output of this model are movement trajectories that result from decisions made by moving individuals interacting with their environment.

**Table 2**: Parameter table of the Correlated Random Walk performed by the agents for different scenarios

| Description | Distribution | Parameters | Value |
| --- | --- | --- | --- |
| Turning angle | Von Mises | location (μ) | 0 |
|  |  | Kappa (ϰ) | 4 |
| Step Length | Exponentiated Weibull Distribution | scale (λ) | 0 |
|  |  | Shape (ϰ) | 6 |
| Selection coefficient  (A.a, A.b) | / | Physical Environment Beta (β⁠) | 100 |
| Selection coefficient  (B) | / | Physical Environment Beta (β⁠) | 10 |
| Selection coefficient  (C) | / | Physical Environment Beta (β⁠) | 0 |
| Selection coefficient  (D) | / | Individual Beta (β⁠) | 4 |

*Adaption, Objectives, Learning, and Prediction*.

None of these design concepts have been used.

*Sensing*. Agents sense other agents and their physical environment within movement constraints.

*Interaction*. One of the agents will be attracted to the other agent, while the other will not interact with the first one. Both agents can also interact with their physical environment. Both interactions can be turned on or off.

*Stochasticity*. Randomness is introduced by the initial random location, the turning angle of the agents, as well as during their biased correlated random walk. Indeed, to generate the available steps at each time step the turning angle and the step length are randomly drawn from a Von Mises and gamma distribution respectively with given parameters. Lastly, the true step is randomly drawn from the available steps based on the redistribution kernel.

*Collectives*. The agent is attracted by the other moving agent. This interaction can be disabled.

*Observation*. To imitate what can be observed in an empirical study that relies on telemetry methods for collecting the movement data, we use the “virtual ecologist approach” (Zurell et al. 2010), i.e. we sample the coordinates of each individual species and individual ID at each time step.

1. Initialization

The position of both agents is randomly chosen within the landscape. Time is modelled as discrete steps. Both agents are also given an initial movement turning angle drawn from a Von Mises distribution. At the beginning of each model run we set up the landscape consisting of 1000 x 1000 cells. The agents are either attracted to the attributes of the environment, do not interact at all with the attributes of the environment but avoid the barriers in the landscape, or only interact with the other agent.

Input data

The model does not use any input data.

1. Sub models

## Movement

Both agents perform an exploratory movement characterized by a correlated random walk defined by their turning angle and step length. The turning angle of the facing direction follows the Von Mises distribution (Codling et al. 2010). The step length is randomly drawn from the exponentiated Gamma distribution (Table 2). The parameters for these two distributions are fixed (Table 2). A value of ϰ = 4 for the Von Mises distribution was chosen to generate a movement with a tendency to move straight forward (Mardia and Jupp 2010). The step length ranges from 0 and a maximum of approximately 4.5. The mean is around 0.9. The agents will not leave the landscape since the available steps generated outside of the landscape are dropped.

## Interactions with other agents

Scenario D: One of the two agents will be constantly attracted to the other agent (bias towards the position of the other individual), mimicking group movement of animals forming herds. The other agent will be neutral and will not interact with the first agent. It will thus decide where to move in the landscape, mimicking the alpha animal in a herd. This leader agent will not interact with the attributes of the landscape and will thus move according to a correlated random walk with specific movement parameters (Table 2).

## Interactions with the physical world

Scenario A & B: Both agents interact with their physical world but do not interact with each other (bias towards the attributes of the landscape). Within the movement limits (turning angle and step length) the agent will select for the attributes of the physical environment. There is a linear selection strength towards these values, the higher the values of the physical environment the stronger the attraction. Both agents will be strongly attracted towards areas with a high density of high-quality environmental attributes.

Scenario C: Both agents move in a highly fragmented landscape with matrix cells (acting as barriers) in which the agents do not move. For each simulated step the generated available steps that fall within the barriers are dropped and therefore the agents will never move into these structures and thus indirectly avoid them. The agents are not attracted to the physical world and do not interact with the other agents. Within the movable space, both agents move according to a correlated random walk with strong directional persistence to mimic dispersing or commuting animals.
